# Armored Droplets as Soft Nanocarriers for Encapsulation and Release under Flow Conditions

**DOI:** 10.1021/acsnano.1c00955

**Published:** 2021-07-15

**Authors:** François Sicard, Jhoan Toro-Mendoza

**Affiliations:** † Department of Physics and Astronomy, University College London, WC1E 6BT London, U.K.; ‡ Department of Chemical Engineering, University College London, WC1E 7JE London, U.K.; ¶ Centro de Estudios Interdisciplinarios de la Fisica, Instituto Venezolano de Investigaciones Cientificas, Caracas 1020A, Venezuela

**Keywords:** smart materials, nanocarrier, Pickering emulsions, flow-assisted encapsulation, dissipative particle dynamics

## Abstract

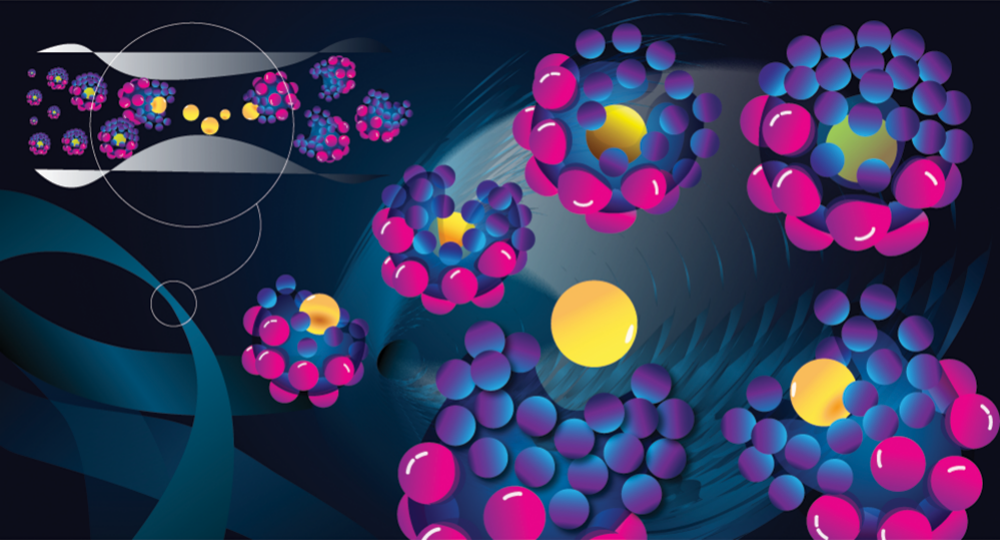

Technical challenges in precision medicine and environmental remediation create an increasing demand for smart materials that can select and deliver a probe load to targets with high precision. In this context, soft nanomaterials have attracted considerable attention due to their ability to simultaneously adapt their morphology and functionality to complex ambients. Two major challenges are to precisely control this adaptability under dynamic conditions and provide predesigned functionalities that can be manipulated by external stimuli. Here, we report on the computational design of a distinctive class of soft nanocarriers, built from armored nanodroplets, able to selectively encapsulate or release a probe load under specific flow conditions. First, we describe in detail the mechanisms at play in the formation of *pocket-like* structures in armored nanodroplets and their stability under external flow. Then we use that knowledge to test the capacity of these pockets to yield flow-assisted encapsulation or expulsion of a probe load. Finally, the rheological properties of these nanocarriers are put into perspective with those of delivery systems employed in pharmaceutical and cosmetic technology.

Considerable attention has been devoted to the design, characterization, and development of nanocarriers built on smart materials due to their potential in targeted-oriented active molecule delivery in biological environments^1^ and their active role in environmental remediation strategies.^2^ This includes soft smart material and soft robotic technology platforms that can be sufficiently miniaturized and demonstrate flexible and adaptative architecture in complex ambients.^3,4^ Materials that fall into this category include but are not limited to structured liquids,^3^ shape-memory alloys and polymers,^5^ stimulus-responsive hydrogels,^6^ and carbon nanotubes.^7^

The morphological and physicochemical properties of soft nanomaterials provide advantages that enable the design of autonomous nanoscale devices that can more closely mimic or interact with biological structures with stimulus-responsive characteristics. In this context, emulsions stabilized with nanoparticles (NPs), also know as Pickering emulsions,^8^ offer real advantages in a wide range of fundamental^9−13^ or industrial and medical applications.^14−16^ They can serve as a template for autonomous platforms that are able to change their shape and adopt different functionalities as a result of morphological transformation obtained *via* interfacial self-assembly and cross-linking,^17,18^ dynamical control of the NP distribution with electric or magnetic fields,^19−26^ or dynamical control of the fluidic environment,^27,28^ among other techniques.

NP-coated droplets have been intensively used as drug-delivery vehicles in topical medication,^29^ where their surfactant-free character makes them attractive for different applications since surfactants often produce adverse effects, such as irritation and hemolytic disturbances.^30,31^ They can serve as ideal compartments for reactions catalyzed by NPs attached at the oil–water interfaces^32−35^ and can be used in bacterial recognition technologies.^36,37^ Another important and useful advantage of NP-coated droplets over conventional surfactant-stabilized systems is their enhanced stabilization against coalescence^38^ and their smaller environmental footprint.^27^

While tremendous progress has been made in highly controlled particle-based microfluidic technology^39,40^ and NP assembly at fluid interfaces,^41,42^ the inherent limitation in experimental resolution eludes direct access to local observables, such as the particles’ three-phase contact angle distribution and the details of the particles’ interfacial network, which present complex geometries.^43^ This information can be accessed by numerical simulations.^38,44−46^ Predicting and controlling this interfacial arrangement under external stimuli to advance the design of the next generation of smart nanocarriers, incorporating soft-matter architectures for precision medicine, is even more challenging.

The recent experimental report by Rozynek *etal*.^12^ on controlled deformation of micron-sized Pickering droplets using electric fields encouraged us to propose strategies for design, analogous to coated nanodroplets under flow. To further contribute in that direction, we report here on the computational design of a distinctive class of soft nanocarriers inspired by Pickering nanoemulsion systems, which can have potential in targeted-oriented active molecule encapsulation and release under specific flow conditions. Those conditions are consistent with the high-shear regime of spreading topical medication on the skin and the transport of targeted carriers in pathological alterations of the vascular system, both of them being successfully reproduced in microfluidic devices.^47−49^ Dissipative particle dynamics (DPD) is employed as a mesoscopic simulation method^50^ with two aims: (1) to describe in detail the formation of *pocket-like* structures in NP-coated nanodroplets and their stability under specific flow conditions and then (2) to test the capacity of the formed pockets to encapsulate or expel a probe load.

Water nanodroplets coated with spherical NPs with different diameters and immersed in an organic solvent are considered. The coating is formed by Janus NPs, *i*.*e*., particles whose surface shows two distinct wetting properties on each half,^51,52^ whose initial three-phase contact angles result in maximum adsorption energy at the fluid–fluid interface.^53^ We first characterize the interfacial properties of the system (three-phase contact angles and diffusion coefficient of the individual NPs, decrease of interfacial tension). We study the role of the NP surface coverage and the interfacial mechanisms at play in the surface mechanical instabilities responsible for the deformation of the armored nanodroplets, when the volume of the droplet is reduced. In particular, we observe in detail the formation of crater-like depressions with selective geometry, which can structurally favor the loading of a probe load. We then investigate the dynamical response of these *pocket-like* structures subjected to homogeneous shear flow using nonequilibrium simulations and the SLLOD equations of motion. Under specific conditions, we observe the formation of long-lived anisotropic structures, characteristic of a jammed particle coverage at the liquid–liquid interface. Furthermore, we examine the capacity of the system to control the flow-assisted encapsulation or expulsion of a probe load, which depends on the interplay between NP surface coverage, the level of buckling, and the shear flow conditions. Finally, we discuss the plausibility of applying the latter predictions to systems in the micron scale, of interest but not limited to pharmaceutical and cosmetic technology.

## Results and Discussion

### System Characteristics

**Figure 1:**
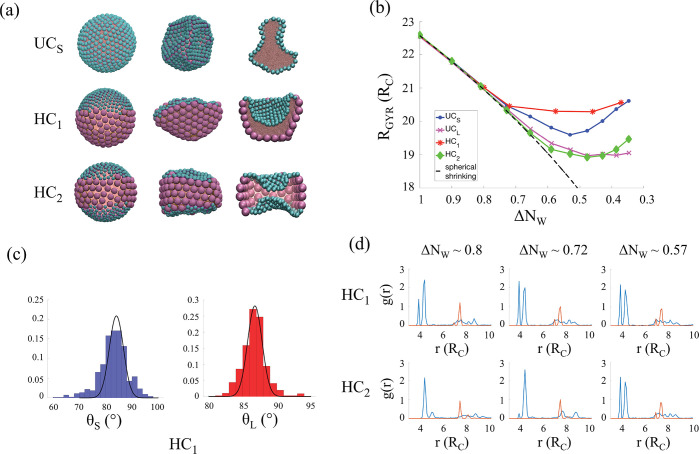
Formation of pocket-like structures. (a) Simulation snapshots representing the initial (left) and final (center) water in oil droplets armored with different nanoparticle surface coverages, obtained from the evaporation process: uniformly covered droplets with small NPs (UC_S_) and heterogeneously covered droplets with either each hemisphere covered with small or large NPs (HC_1_) or three distinct layers made of small–large–small NPs (HC_2_). The cross-sectional view of each system is also shown (right). Cyan and purple spheres represent the small and large Janus NPs, respectively. The detailed structure of the NPs is shown in Figure S1a in the SI. Pink spheres represent water beads. The oil molecules surrounding the system are not shown for clarity. (b) Evolution of the radius of gyration, *R*_GYR_, of UC_S_, UC_L_, HC_1_, and HC_2_, as a function of the dimensionless parameter Δ*N*_W_ = *N*_W_/*N*_W_^(0)^. *N*_W_ represent the number of water beads that remain in the droplet after each removal, and *N*_W_^(0)^ is the initial number of water beads. The statistical errors are estimated as one standard deviation from the average obtained for equilibrated trajectories and are always smaller than the symbols. The dashed lines represent the spherical-shrinking regime defined as *R*_GYR_ ≈ (Δ*N*_W_)^1/3^. (c) Three-phase contact angle distribution of small (blue) and large (red) NPs for HC_1_ after the last pumping/equilibration iteration (Δ*N*_W_ ≈ 0.35). (d) Evolution of the radial distribution function, *g*(*r*), with *r* the distance between the center of the NPs, of small (blue) and large (red) NPs for HC_1_ (b) and HC_2_ (c).

We first give the characteristics and assess the interfacial properties of the NP-coated droplets considered in this work. In Figure 1a we show representative snapshots of the water emulsion nanodroplets in an organic solvent (decane) stabilized with Janus NPs. The scaled temperature in the DPD framework is equivalent to 298.73 K. The details of the numerical parametrization and NP structures are given in the Methods section and the Supporting Information (SI). The configurations differ by the size of the NPs and the characteristics of the surface coverage. We consider small (S) and large (L) NPs with diameters *d*_S_ ≈ 2.2 nm and *d*_L_ ≈ 4.5 nm, whose diffusion coefficients measured on a planar decane/water interface are *D*_*S*_ ≈ (4.7 ± 3.1) × 10^–7^ cm^2^ s^–1^ and *D*_L_ ≈ (1.8 ± 0.7) × 10^–7^ cm^2^ s^–1^, respectively (see Methods and Figure S1c in the SI).

The NPs are originally located at the surface of the emulsion nanodroplets of diameter *d*_D_ ≈ 45 nm. Similar NP surface coverage ϕ ≈ 0.8 is considered on the armored nanodroplets.^44,54^ This yields similar initial three-phase contact angles θ_S_ ≈ 84.1 ± 2.7° and θ_L_ ≈ 86.8 ± 1.1° for the small and large NPs, respectively (see Methods and Figure S1b in the SI), in qualitative agreement with simulations^38,55,56^ and experimental observations.^57^ From the error bars estimated, it is observed that the small NPs are more sensitive to thermal fluctuations at the interface compared to the large ones, characteristic of the increase of the adsorption energy with the particle radius.^58−60^ We also measure the decrease of the interfacial tension, Δγ_S_ and Δγ_L_, for small and large NPs at planar interfaces for similar NP surface coverage (see Methods). We obtain Δγ_S_ = γ_0_ – γ_S_ ≈ 5.1 mN·m^–1^ and Δγ_L_ = γ_0_ – γ_L_ ≈ 2.2 mN·m^–1^, with γ_0_ ≈ 51.7 mN·m^–1^ being the interfacial tension for a planar decane/water interface,^46,56^ and γ_S*,*L_ the interfacial tension when the interface is covered with small or large NPs, respectively. In particular, large NPs have less effect on the reduction of the interfacial tension and are less diffusive than smaller ones, in qualitative agreement with simulations^60^ and experimental observations.^61^ As we will see in the following sections, the difference in mobility and size of the NPs play a key role in the pocket formation.

### Formation of Pocket-like Structures

To obtain the desired *pocket-like* morphology at the surface of the nanodroplets, which would eventually favor the loading of a probe load, the volume of the droplets is systematically reduced, by iteratively pumping a small constant proportion of water molecules out of the droplets and letting the systems equilibrate between each iteration (see details in the SI). The systems present dimples and cups at the droplet interface followed by the formation of crater-like depressions, characteristic of the buckling instability.^44,62,63^ This process is physically equivalent to a process of solubilization of the dispersed phase into the solvent.^44,62^ We arbitrarily stop the pumping when the number of water molecules constituting the droplets reaches the value Δ*N*_W_ = *N*_W_/*N*_W_^(0)^ ≈ 0.35, where *N*_W_^(0)^ and *N*_W_ are the initial number of water beads and the number of water beads remaining in the droplets, respectively.

In Figure 1b, we show the evolution of the radius of gyration of the emulsion nanodroplets, *R*_GYR_, as a function of the dimensionless parameter Δ*N*_W_. We initially consider spherical droplets whose surface is either uniformly covered (UC) with NPs of identical diameter or heterogeneously covered (HC) with NPs of different diameters segregated on each side. Specifically, UC_S_ (respectively UC_L_) is solely covered with small (respectively large) NPs, as shown in Figure 1a. HC_1_ and HC_2_ have each hemisphere covered with small and large NPs or three distinct layers made of small–large–small NPs, respectively (*cf*. Figure 1a). When Δ*N*_W_ > 0.75, the radius of gyration of the four systems follows a similar evolution, regardless of the NP coverage (UC or HC), characteristic of a spherical-shrinking regime, *R*_GYR_ ≈ (Δ*N*_W_)^1/3^ (dashed line in Figure 1b). When Δ*N*_W_ < 0.75, the systems follow different transitions from spherical shrinking to buckling, depending on the characteristics of the NP interfacial packing originating from the difference in surface coverage.^64^ This transition happens when the NP monolayer becomes close to its maximum packing, as observed with the evolution of the radial distribution function *g*(*r*), with *r* being the distance between the center of the NPs, shown in Figure 1d and Figures S2 in the SI. The radial distribution function (rdf) has been intensively used to study the evolution of the ordering of NP aggregates in Pickering emulsions, consistent with experimental observations. In particular, UC_S_ and UC_L_ show different morphological evolutions when Δ*N*_W_ decreases, with UC_S_ entering the buckling regime at larger Δ*N*_W_ than *UC*_L_, in qualitative agreement with the numerical work of Gu *etal*.^63^ Finally, below Δ*N*_W_ ≈ 0.45, *R*_GYR_ increases as the droplets can be described as half-sphered.

The structures of the armored nanodroplets obtained after the last pumping/equilibration iteration are shown in Figure 1a (central panel). Visual inspection shows different folding morphologies, depending on the characteristics of the NP coverage. Unlike UC, where crater-like depressions form evenly at the interface under the effect of a compressive surface stress, we observe the formation of well-localized crater-like depressions in heterogeneously covered systems (HC_1_ or HC_2_). This latter evolution depends on the localization of the interfacial areas covered with small or large NPs. Notably, we observe the crater-like depressions form in the interfacial areas covered with the smallest NPs, where maximum packing of the interfacial network is achieved quicker and the interfacial tension is lower than those measured for larger NPs.

The properties of the interfacial layers are quantitatively assessed *via* the analysis of the distribution of the three-phase contact angles, θ_C_^(S)^ and θ_C_^(L)^, of small and large NPs, respectively. As shown in Figure S1b in the SI, θ_C_^(S)^ and θ_C_^(L)^ follow Gaussian distributions in the initial configurations (Δ*N*_W_ ≈ 1), where the shape of the droplets is spherical. When the volume of UC_S_ and UC_L_ is reduced, θ_C_^(S)^ and θ_C_^(L)^ uniformly evolve from Gaussian to skewed unimodal distributions, in line with previous work.^44^ The values of the respective means, μ_*S*_ and μ_L_, and standard deviations, σ_S_ and σ_L_, for small and large NPs, respectively, are shown in Table 1. Whereas the contact angle distributions show a single peak centered at the same value as the one measured for the initial configuration, σ_S_ and σ_L_ show significant variations when the volume of the droplets is reduced, characteristic of the skewness of the distribution and the decrease of the NP–NP distance (*cf*. Figure S2 in the SI). When the volume of HC_1_ and HC_2_ is reduced, on the other hand, we observe significant differences in the evolution of the distributions of θ_C_^(S)^ and θ_C_^(L)^, due to the heterogeneity in NP size and surface coverage. In particular, the distribution of θ_C_^(L)^ is similar to the one measured in the initial configuration, while the distribution of θ_C_^(S)^ shows large variability, similar to the one measured in UC_S_, during the buckling transition, originating from the difference in packing of the monolayer at the droplet interface, as shown in Figure 1c.

**Table 1:** Measure of the Mean (μ) and Standard Error (σ) of the Three-Phase Contact Angle Distribution in UC and HC Droplets in the Initial (Δ*N*_W_ ≈ 1.0) and Final (Δ*N*_W_ ≈ 0.35) Configurations

Δ*N*_W_		UC_S_	UC_L_	HC_1_	HC_2_
1.0	(S)	84.1 ± 2.7°		84.1 ± 2.7°	84.1 ± 2.7°
	(L)		86.8 ± 1.1	86.8 ± 1.1°	86.8 ± 1.1°
0.35	(S)	82.9 ± 5.9°		82.8 ± 6.0°	82.4 ± 6.4
	(L)		83.6 ± 9.9°	86.7 ± 1.9°	87.0 ± 1.8

From the analysis above, we showed that the formation of well-localized *pocket-like* structures at the surface of the NP-coated droplets can be selectively controlled *via* the solubilization/evaporation of the disperse phase and with the use of heterogeneous NP layer coverage at the surface of the droplets. This evolution results from the interplay between thermodynamic and steric effects at the liquid/liquid interface, with the difference in NP adsorption energy, interfacial tension, and interfacial packing, between small and large NPs. Moreover, the formation of the *pocket-like* depressions is observed to be primarily controlled by the reorganization of the small NPs at the surface of the heterogeneously coated droplets. The characteristic of the NP interfacial layer can be further tuned to control the size and the number of *pocket-like* depressions that can form in each hemisphere of the NP-coated droplets.

### Dynamical Response under Shear Flow

**Figure 2:**
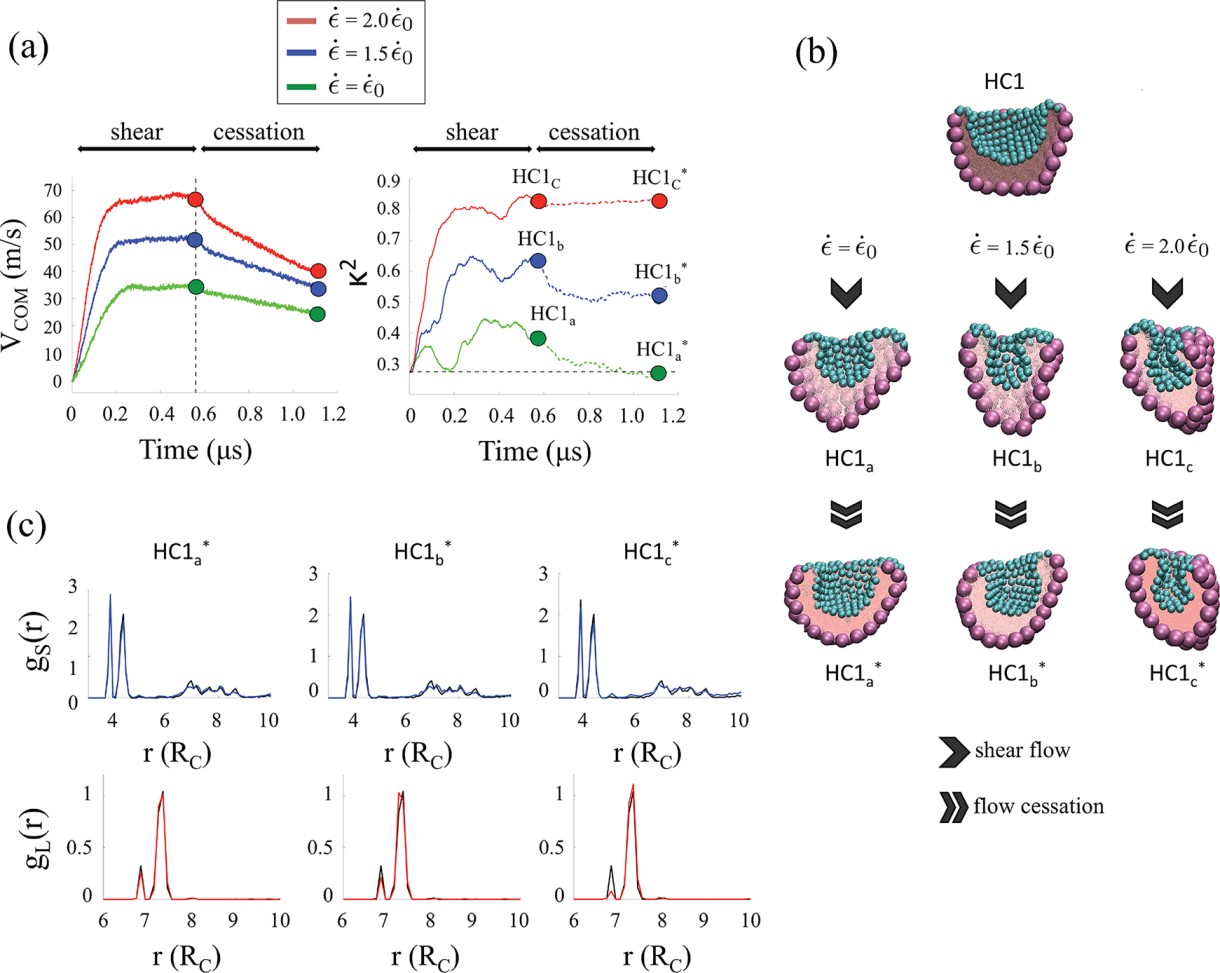
Dynamical response under shear flow. (a) Temporal evolution of the velocity center of mass *V*_COM_ and the relative shape anisotropy κ^2^ of HC_1_ subjected to shear flow and after abrupt shear cessation for three different values of the shear rate ϵ̇. The shear flow is continuously applied for a time duration Δ*t* ≈ 0.6 μs before it is abruptly stopped and the structure relaxes for another Δ*t* ≈ 0.6 μs. (b) Representative snapshots of the armored nanodroplets obtained just before the flow cessation (*t* ≈ 0.6 μs) and at the end of the simulation (*t* ≈ 1.2 μs). Cyan and purple spheres represent the small and large Janus NPs, respectively. The detailed structure of the NPs is shown in Figure S1a in the SI. Pink spheres represent water beads. The oil molecules surrounding the system are not shown for clarity. (c) Radial distribution function, *g*_S_(*r*) and *g*_L_(*r*), with *r* being the distance between the center of the NPs, of small (blue) and large (red) NPs for HC1_*a*,*b*,c_^*^. The corresponding radial distribution functions measured before the shear flow is applied (HC1) are shown in black color for comparison.

Thereafter, we investigate the structural response of the *pocket-like* structures, as obtained from volume reduction of the armored droplet, subjected to shear flow of the surrounding fluid using the SLLOD algorithm^65,66^ coupled with Lee–Edwards periodic boundary conditions^67^ (see Methods). We focus our analysis on HC_1_, whose structural morphology is more likely to yield better loading of a probe load (*cf*. Figure 1a). The minimum value for the shear rate, ϵ̇_0_ ≈ 0.9 ns^–1^, is set to the value for which the initial structure starts showing significant deformations. The system is first stressed under a constant shear rate, ϵ̇ = α × ϵ̇_0_, along the *x*-axis for a time duration Δ*t* ≈ 0.6 μs, with the parameter α = 1.0, 1.5, and 2.0. The length of the simulation is chosen to be sufficiently long for the velocity center of mass of the droplet, *V*_COM_, to level off to a plateau whose value matches that obtained from the stationary velocity profile of laminar flow, *V*_COM_ = ϵ̇ × *L*_*y*_/2, with *L*_*y*_ ≈ 77 nm, the size of the simulation box along the *y*-direction (*cf*. Figure 2a). The flow is then abruptly halted and the dynamical stability of the nanodroplet is studied for a time duration Δ*t* ≈ 0.6 μs.

Representative snapshots of the structural morphology of the armored nanodroplets obtained after *t* ≈ 0.6 μs and *t* ≈ 1.2 μs, identified in Figure 2a, are shown in Figure 2b. Visual inspection shows different morphologies depending on the intensity of the shear rate and the relaxation of the system. The changes in structural morphology are quantitatively assessed with the measure of the relative shape anisotropy parameter, κ^2^, which reflects both the symmetry and dimensionality of the system^68,69^ (see Methods). As shown in Figure 2a (right panel), we observe the increase of κ^2^ for a relatively short time until it levels off to a plateau when the velocity profile of the laminar fluid becomes stationary, and its value depends on the intensity of the shear rate. In Figure 2b and Figure S3a in the SI, we observe the increase of κ^2^, associated with the elongation of the droplet along the deformation axis *x* and with the squeezing of the crater-like depression along the orthogonal *z*-direction. (HC1_*a*,*b*,*c*_).

When the flow is abruptly halted at *t* ≈ 0.6 μs, we observe either the relaxation of κ^2^ toward its initial value (HC1_a_^*^) or the formation of a long-lived anisotropic structure (HC1_*b*,c_^*^), depending on the intensity of ϵ̇. The specificity of the structural morphology of HC1_*b*,c_^*^ can be explained by the formation of a jammed particle layer at the droplet interface, in qualitative agreement with recently reported experimental observations.^28^ To do so, we assess the characteristics of the NP interfacial layer of HC1_*a*,*b*,c_^*^ with the analysis of the three-phase contact angle distribution and the NP radial distribution function of small and large NPs. Within the range of shear rates considered in this work, θ_C_^(L)^ follows a Gaussian distribution of mean μ_L_ ≈ 87.2° and standard deviation σ_L_ ≈ 1.8°, similar to the one measured in both the initial and buckled configurations (*cf*. Figure 1c). θ_C_^(S)^, on the other hand, shows a skewed unimodal distribution with a central peak located at the same value as the one measured for both the initial and buckled configurations. The skewness of the distribution does not depend significantly on the intensity of the shear rate within the standard errors (*cf*. Figure S3 in the SI).

Most importantly, the radial distribution functions, *g*_S_ and *g*_L_, of small and large NPs, respectively, show different behaviors depending on the size of the NPs, as shown in Figure 2c. Whereas *g*_S_ follows the same evolution as the one measured in HC_1_ before the shear rate is applied, the evolution of *g*_L_ reflects the local reorganization of the layer made solely of large NPs at the droplet interface, as shown with the gradual decrease of its first peak associated with the first coordination sphere, eventually recovering the distribution observed in the initial spherical configuration shown in Figure 1d.

From the nonequilibrium simulations performed above, we showed the ability of the NP interfacial layer to control the structural evolution and dynamical stability of the armored nanodroplets under the application of external homogeneous shear flow of the surrounding fluid. Whereas the formation of the *pocket-like* structures was essentially driven by the reorganization of the small NPs at the liquid/liquid interface, the dynamical evolution under shear flow appears to be driven by steric effects with the local reorganization of the large NPs forming the jammed NP coverage at the droplet interface, which can be selectively tuned with the intensity of the shear rate. These results suggest that the morphology of the *pocket-like* armored droplet could be dynamically adjusted to accommodate probe loads of various sizes, as we investigate in the following.

### Encapsulation and Release of Probe Load

**Figure 3:**
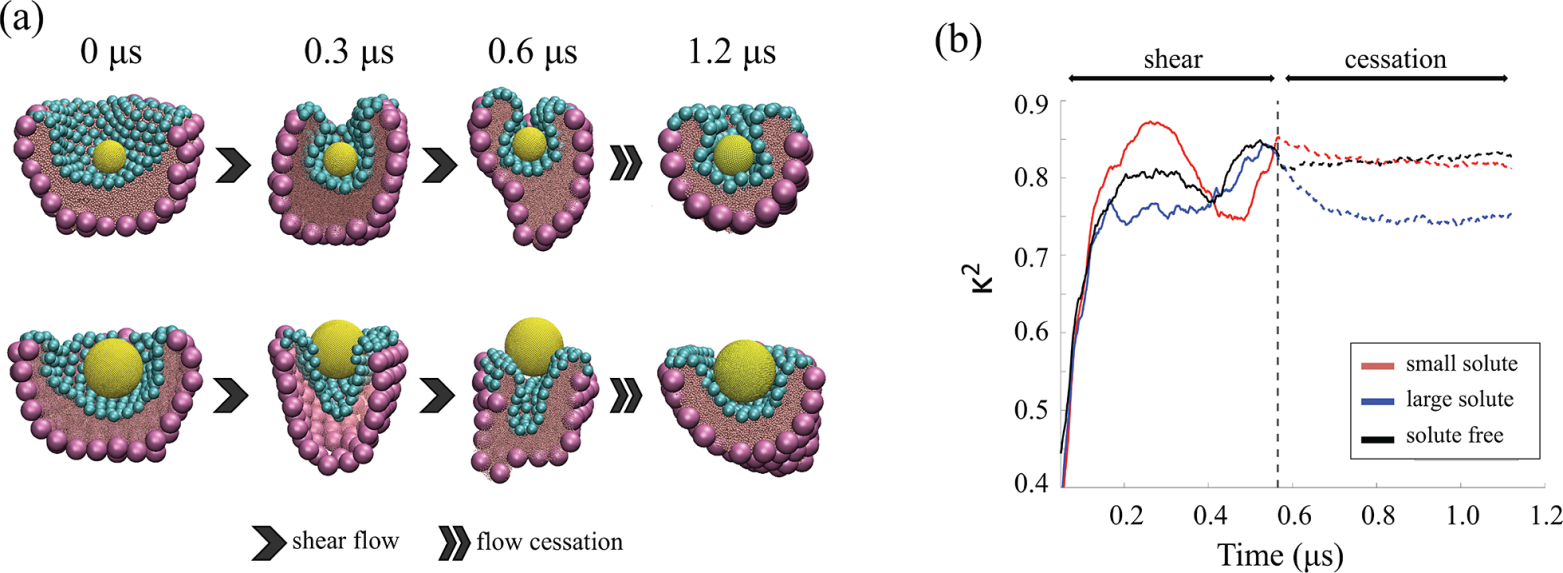
Encapsulation and release of the probe load. (a) Representative snapshots of the structural morphology of HC1 preliminary loaded with small (S) and large (L) hydrophobic spherical solute of diameter *d*_S_ ≈ 7.7 nm (top panel) and *d*_L_ ≈ 15.4 nm (bottom panel), respectively, at different simulation stages. Cyan and purple spheres represent the small and large Janus NPs, respectively. The detailed structure of the NPs is shown in Figure S1a in the SI. Pink and gold spheres represent water and solute beads. The oil molecules surrounding the system are not shown for clarity. (b) Representative temporal evolution of the relative shape anisotropy, κ^2^, of HC_1_ loaded with small and large solute, subjected to shear flow (ϵ̇ = 2 × ϵ̇_0_) and after abrupt shear cessation. The shear flow is continuously applied for a time duration Δ*t* ≈ 0.6 μs before it is abruptly stopped and the structure relaxes for another Δ*t* ≈ 0.6 μs. The evolution of κ^2^ for the free system is shown for comparison.

Our results so far allow us to address our second aim of investigating the dynamical response of the system under shear stress, when the buckled armored nanodroplet is first loaded with a probe load, as shown in Figure 3a. We determine the ability of HC1_c_ to lead to the encapsulation or release of the solutes under flow conditions identical to those studied in the free configuration. We consider the largest shear rate, ϵ̇ = 2 × ϵ̇_0_ ≈ 1.8 ns^–1^, which shows the strongest morphological deformation of the system, as shown in Figure 2a (right panel). Two spherical hydrophobic solutes are considered: one small (*S*_S_) with radius *r*_S_^(s)^ ≈ 4 nm and one large (*S*_L_), with a radius *r*_L_^(s)^ ≈ 8 nm, respectively. The sizes of *S*_S_ and *S*_L_ are specifically chosen so that they can be initially loaded in the crater-like depression formed at the interface of HC1, obtained after the last removal of water (*cf.*Figure 1a). *S*_S_ and *S*_L_, however, differ in their ability to eventually fit or not in HC1_c_ when the shear stress is applied. The characteristics of the spherical solutes in the DPD framework are given in the Methods section.

The system is first stressed under constant shear rate along the *x*-axis for a time duration Δ*t* ≈ 0.6 μs, sufficiently long to observe the flow-assisted encapsulation or release of the small and large solutes, respectively. The flow is then abruptly halted, and the relaxation of the system is studied for a time duration Δ*t* ≈ 0.6 μs. In Figure 3a we show representative snapshots of the systems loaded with the two spherical solutes, *S*_S_ and *S*_L_, at different simulation stages. When the solute is sufficiently small, the particle-laden interface folds inward under surface stress, leading to the encapsulation of the solute. When the solute is sufficiently large, however, the crater-like depression cannot accommodate the solute when the system is stressed. Therefore, *S*_L_ is progressively expelled from the pocket following the narrowing and elongation of the nanodroplet. As the flow is abruptly halted, the armored nanodroplet relaxes its structural morphology, accommodating the solute load inside the residual pocket, regardless the size of the load.

The evolution of the structural morphology of the loaded nanodroplets is quantitatively assessed with the estimation of the relative shape anisotropy, κ^2^, as shown in Figure 3b. In particular, we compare the average value of κ^2^ in the stationary regime, *i*.*e*., 0.2 μs ≤ *t* ≤ 0.6 μs, defined as , along with the relative change δκ^2^ = |κ^2^(*t* = 1.2 μs) – κ^2^(*t* = 0.6 μs)|/κ^2^(*t* = 0.6 μs), measured between the beginning (*t* = 0.6 μs) and the end (*t* = 1.2 μs) of the relaxation period. As shown in Table 2, the values of ⟨κ^2^⟩ estimated in the free and loaded configurations do not differ significantly within the standard errors, suggesting the pocket-like nanodroplet passively encapsulates or expels the small and large solutes, respectively, under the flow conditions and solute characteristics considered in this work. When the flow is abruptly halted, on the other hand, we observe the relaxation of the system, which accommodates the solute load inside the residual pockets. During this process, the relaxation of the structural morphology of the loaded nanodroplets differs from the solute-free configuration, as quantified with the relative change δκ^2^ in Table 2, in qualitative agreement with the visual inspection in Figure 3a.

**Table 2:** Estimation of the Average Value of κ^2^ When the System Reaches a Stationary State under Flow Conditions, ⟨κ^2^⟩, and the Relative Change δκ^2^ between the Beginning and the End of the Relaxation Period^a^

	free	*S*_*S*_	*S*_*L*_
⟨κ^2^⟩	0.81 ± 0.02	0.79 ± 0.04	0.80 ± 0.05
δκ^2^	1.2 ± 0.3%	4.4 ± 1.6%	6.4 ± 4.5%

a Uncertainties are determined by considering three replicas of the systems and calculating the standard error.

The results above highlight the ability of the buckled armored droplets to dynamically adjust their morphology under external stress and adapt their response to probe loads with definite size and shape. This versatility is essential for the design of soft nanocarriers capable of adapting their physical properties when exposed to an external stimulus, such as mechanical stress, in complex ambients, as discussed in the next section.

### Perspectives in Delivery Technology

The flow-assisted encapsulation and release of load probes in armored nanodroplets reported so far can be extended to systems of larger dimensions under conditions similar to those expected in the high-shear regime of spreading topical medication on the skin (such as creams and ointments) and the transport of targeted carriers in pathological alterations of the vascular system (such as venous or arterial thrombosis). These predictions would depend on the original dimension of the spherical droplet along with the initial NP surface coverage and the NP dimension to droplet size ratio, which would affect the surface area to volume ratio of the system and the average surface pressure of the particle-laden interface,^63^ respectively.

To extend our results, the flow properties of the system are analyzed with two essential control parameters, *i*.*e*., the Weber number (*We*) and the Ohnesorge number (*Oh*), commonly used in microfluidic^70,71^ and droplet formation.^72^ The Weber number, *We* = ρ_o_*v*^2^*d*_D_/γ, represents the ratio of the disrupting inertial force to the restorative surface tension force, where ρ_o_ and *v* are the density and the relative velocity of the ambient fluid (decane oil) and *d*_D_ and γ are the diameter and the interfacial tension of the droplet, respectively. The Ohnesorge number, , represents the relative importance of the viscous force to the inertial and surface tension forces, where μ_W_ and ρ_W_ are the dynamic viscosity and the density of the water droplet, respectively. From the calculation of *Oh*, one can define the critical Weber number, *We*_C_ = 12(1 + 1.5 × *Oh*^0.74^), which corresponds to the minimum Weber number for a droplet to exhibit breakup modes.^73^ Given γ ≈ 51.7 mN·m^–1^ is the interfacial tension for a planar decane/water interface,^46,56^ ρ_W_ ≈ 1000 kg·m^–3^ and ρ_o_ ≈ 726 kg·m^–3^ are the density of water and decane oil, respectively, *v* ≈ 50–70 m·s^–1^ is the stationary velocity of the laminar flow (*cf*. Figure 3a), μ_W_ = 8.9 × 10^–4^ Pa·s is the dynamic viscosity of water, and *d*_D_ ≈ 40 nm is the droplet diameter obtained from the measure of *R*_GYR_ (*cf*. Figure 2a), we obtain *Oh* ≈ 0.6, *We*_*C*_ ≈ 25, and *We* ≈ 1.4–2.8, indicating the armored droplets considered in the flow-assisted encapsulation and release processes are outside their breakup regime.^74^

Now, based on the estimation of the Weber number, we extend our predictions to the high-shear regime of spreading water-in-oil/oil-in-water emulsion-based products. Given the relation *v* ≈ ϵ̇ × *L*_⊥_ with *L*_⊥_ being the dimension of the system orthogonal to the flow direction, we obtain *We* ≈ ρ_o_ϵ̇^2^*L*_⊥_^2^*d*_D_/γ. Considering the average thickness of a cream *L*_⊥_≈ 1 cm and representative shear rates ϵ̇ ≈ 10^2^–10^3^ s^–1^,^75,76^ we obtain the characteristic dimension of the emulsion droplet *d*_D_ ≈ 1–100 μm, corresponding to the minimal droplet size to observe the encapsulation or release mechanism, in agreement with the range of characteristic droplet sizes commonly used in topical pharmaceutical products.^76,77^

Either by skin adsorption or other intake paths, targeted carriers can reach the bloodstream as required. The complexity of the flow scenarios present in the circulatory system defies the full description of the behavior of our model carrier once entering the body. However, it is possible to put our predictions into perspective with the transport of our model carrier in the vascular subsystem, in particular in the pathological flow conditions encountered in venous or arterial thrombosis.^78^ The fluid properties of the hepatic artery (representative of a large artery) in nonpathological conditions have a characteristic dimension *L*_⊥_≈ 5 mm and shear rate ϵ̇ ≈ 500 s^–1^.^79^ A pathological flow, on the other hand, can be defined as where the blood reaches shear rates ϵ̇ > 5000 s^–1^, resulting, for example, from pathological clotting of blood within the lumen of a vessel.^80^ Considering ρ_blood_ ≈ 1060 kg·m^–3^ and γ ≈ 42 mN·m^–1^ as representative values of the average density and interfacial tension (against fluorocarbon) of the blood fluid,^81^ along with the narrowing of the pathological vessel, *L*_⊥_ → *L*_⊥_/2, we obtain *d*_D_ ≈ 500 nm for the minimal droplet dimension under the condition of a hepatic artery with pathological alterations to observe the encapsulation or release mechanism. For comparison, we obtain *d*_D_ ≈ 10 μm for the minimal droplet dimension in the conditions of the normal hepatic artery, in the range of sizes characteristic of leucocytes and red blood cells.^82^

**Figure 4:**
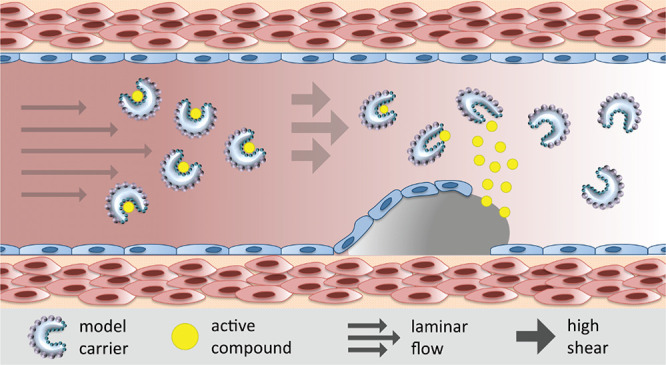
Perspective in delivery technology. Schematic representation of the potential application of the model nanocarrier in the high-shear regime encountered in hepatic artery thrombosis. In that scenario, the decrease in the channel diameter increases the shear rate, promoting the release of the compound (yellow). The cross-sectional view of the model nanocarrier is shown for convenience.

This analysis suggests that the process of targeted delivery of active compounds (such as antithrombotic agents) can be selectively controlled with the size of the model nanocarrier, as shown in Figure 4, depending on the dimension of the compound. For instance, cargo nanodroplets of dimension ∼500 nm would only release the therapeutic payloads they carry once they reach pathological alterations where the diameter of the artery significantly decreases, increasing, consequently, the shear rate of the surrounding fluid. Finally, the important advances in the development of microfluidics devices to closely reproduce cardiovascular systemic conditions in the presence of cells and tissues^47−49^ depict a framework adequate to test and refine our predictions.

## Conclusions

The numerical simulations discussed above allowed us to explore the design and dynamical stability of a distinctive class of soft nanocarriers inspired by Pickering nanoemulsions, which can have potential in targeted-oriented active molecule encapsulation and release under specific flow conditions. The success in controlling the creation of crater-like depressions on the surface of Pickering micron-sized droplets recently reported by Rozynek *etal*.^26^ demonstrates the plausibility of our predictions as well as the dynamics here presented. The interplay between the evolution of the structural morphology of the armored nanodroplets and the organization of the NP interfacial network, when the volume of the system is reduced, is in qualitative agreement with experimental observation.^62^ We showed that finite-size NPs can strongly affect the droplet shape with the formation of *pocket-like* depressions, which can structurally favor the loading of a probe load.

We then focused our analysis on the dynamical tuning of the NP distribution at the surface of the armored nanodroplets under external shear stress, which is more representative of biological ambients. The dynamical response of specifically designed *pocket-like* nanodroplets under different shear flow conditions exhibited the formation of long-lived anisotropic structures, characteristic of a jammed particle coverage at the liquid–liquid interface, associated with the dynamical rearrangement of the NP interfacial network. Most importantly, the ability of *pocket-like* nanodroplets to encapsulate or release spherical solute loads located inside the crater-like depression, during their transport under shear-flow conditions, was validated.

Our predictions on the flow-assisted encapsulation and release of load probes in armored nanodroplets were extended to systems in the micron scale encountered in pharmaceutical and cosmetic technologies. Noticeably, we demonstrated that the mechanism reported in our work could be at play at larger scales, such as those encountered in the high-shear regime of spreading creams and ointments on the skin and the transport of targeted carriers in pathological alterations of the vascular system. As an example, we put the physical properties of our model carrier into perspective within the conditions encountered in pathologies of the hepatic artery, where the formation of a blood clot inside the blood vessel can obstruct the flow of blood through the circulatory system, increasing the hemodynamic shear stress and the risk of bleeding complications. In particular, hepatic artery thrombosis can be a very serious complication of liver transplantation, with mortality in children as high as 70%.^83^ Hence, it is essential to develop distinctive means to control the process of targeted delivery of antithrombotic agents in the vascular system.

The physical insights discussed here provide a deeper understanding on the potential role played by nanoparticle-stabilized emulsions in the biomimetic design of hybrid soft materials for targeted-oriented active load delivery. This information could be useful for a variety of applications including the design of pharmaceutical carriers for drug delivery and pathogen encapsulation, where knowledge of the rheological properties of the system must be quantitatively assessed. Eventually, our method would allow including specific interactions inside the formed cavity in order to mimic, for example, protein binding pockets or catalytic nanosurfaces. Finer morphological changes and functionalization of the surface of the armored droplets could also be considered using pH- or electro-reponsive NPs, therefore controlling their distribution and physicochemical properties at the liquid/liquid interface with external stimuli.^20−25^

## Methods

### Mesoscopic Framework

The dissipative particle dynamics simulation method^84^ is implemented within the simulation package LAMMPS.^85^ In the DPD simulations, a particle represents a cluster of atoms rather than an individual atom. These particles interact with each other through soft particle–particle interactions. The movement of the particle can be realized by solving the Newton equation of motion:
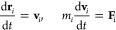
1where *m*_*i*_, **r**_*i*_, **v**_*i*_, and **F**_*i*_ denote the mass, position, velocity, and total force acting on the *i*th particle, respectively. The total force **F**_*i*_ is divided into three parts, the conservative force (**F**_*ij*_^*C*^), dissipative force (**F**_*ij*_^D^), and random force (**F**_*ij*_^R^), and defined as **F**_*i*_ = ∑_*j*≠*i*_(**F**_*ij*_^C^ + **F**_*ij*_^C^ + **F**_*ij*_^C^) with
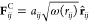
2
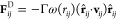
3
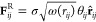
4where **r**_*ij*_ = **r**_*i*_ – **r**_*j*_, *r*_*ij*_ = |**r**_*ij*_|, *r̂*_*ij*_ = **r**_*ij*_/*r*_*ij*_, and **v**_*ij*_ = **v**_*i*_ – **v**_*j*_. The weight function ω(*r*_*ij*_) equals (1 – *r*_*ij*_/*R*_c_)^2^ with a cutoff distance *R*_c_. *a*_*ij*_, Γ, σ, and θ_*ij*_ are the repulsive parameter, friction coefficient, noise amplitude, and Gaussian random variable, respectively. To keep the temperature of the system constant, Γ and σ satisfy the fluctuation–dissipation theorem as σ^2^ = 2Γ*k*_B_*T*, where *k*_B_ and *T* are the Boltzmann and the absolute temperature, respectively.

The system simulated here is composed of water, oil (decane), NPs, and solute molecules. Following previous work,^38,44,46,54^ we choose the degree of coarse graining *N*_*m*_ = 5 with the understanding that one “water bead” (w) represents five water molecules. Within this assumption, the volume of each bead is *V*_bead_ ≈ 150 Å^3^. The scaled density is set to ρ = 3 beads/*R*_c_^3^, where *R*_c_ is the DPD cutoff distance given as  nm. The scaled mass of each bead (oil, water, solute molecule, and NP beads) was set to 1. One decane molecule is modeled as two “oil beads” (o) connected by one harmonic spring of length 0.72*R*_c_ and spring constant 350 *k*_B_*T*/*R*_c_.^86^ The size of the triclinic simulation box (initially orthogonal) is *L*_*x*_ × *L*_*y*_ × *L*_*z*_ ≡ 200 × 100 × 100 *R*_c_^3^, where *L*_*x*_ (respectively *L*_*y*_ and *L*_*z*_) is the box length along the *X* (respectively *Y* and *Z*) direction. Periodic boundary conditions are applied in all three directions. The solute molecules and the NPs are modeled as hollow rigid spheres, as already described in previous work.^38,44,46,54^ The hydrophobic solute molecules are made of nonpolar DPD beads, whereas the NPs contain polar (p) and nonpolar (ap) DPD beads on their surface.^87^ One DPD bead was placed at the NP and solute molecule centers for convenience, as described elsewhere.^54,88^ All types of beads in our simulations have a reduced mass of 1. We maintain the surface bead density on the NPs and solute molecule sufficiently high to prevent other DPD beads (either decane or water) from penetrating the NPs and solute molecules.^88^

The interaction parameters shown in Table 3 are used here. These parameters are adjusted to reproduce selected atomistic simulation results, as explained in prior work.^54^ The interaction parameters between NP polar and nonpolar beads, as well as solute molecule beads, are adjusted to ensure that NPs/NPs and NPs/solute are able to assemble and disassemble without yielding permanent dimers at the water/oil interface.^54^ The scaled temperature was set to 1, equivalent to 298.73 K. The time step δ*t* = 0.03 × τ was used to integrate the equations of motion, where τ is the DPD time constant. As demonstrated by Groot and Rabone,^86^ the time constant of the simulation can be gauged by matching the simulated self-diffusion of water, *D*_sim_, with the experimental water self-diffusion coefficient, *D*_water_ = 2.43 × 10^–5^ cm^2^/s,^89^ calculated as , as shown in previous work.^54^ When *a*_w–w_ = 131.5 *k*_B_*T*/*R*_c_, this results in a time step δ*t* = 5.6 ps.

**Table 3:** DPD Interaction Parameters Expressed in *k*_B_*T*/*R*_c_ Units^a^

	w	o	ap	p	s
w	131.5	198.5	178.5	110	670
o		131.5	161.5	218.5	161.5
ap			450	670	450
p				450	670
s					131.5

a Symbols w, o, ap, p, and s stand for water beads, oil beads, NP nonpolar beads, NP polar beads, and solute beads, respectively.

While the DPD framework satisfies the Navier–Stokes equations in the continuum limit,^84^ the traditional DPD algorithm cannot reproduce the vapor–liquid coexistence of water at the droplet interface.^90^ This is due to the DPD conservative force, which determines the thermodynamics of the DPD system and yields the equation of state^84^
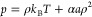
5where *p* is the pressure, ρ is the number density of the DPD beads, *a* is the repulsion strength, and α is a fitting parameter equal to 0.101 ± 0.001 in DPD reduced units.^84^ As shown by Warren,^90^ the DPD system is unstable for *a* < 0, so one is restricted to *a* ≥ 0 and therefore to strictly repulsive (conservative) interactions. This implies that calculations such as the vapor–liquid coexistence and free-surface simulations cannot be attempted. This can be adjusted by considering higher order terms of the density, ρ, in eq 5, *i*.*e*., making the conservative force in eq 2 density dependent.^90^

### Nonequilibrium Simulation

To simulate the response of the system subjected to a homogeneous shear flow, we employ the SLLOD algorithm^65,66^ coupled with the Lee–Edwards periodic boundary conditions,^67^ as implemented in the simulation package LAMMPS.^85^ The SLLOD algorithm modifies the equations of motion in eq 1 as
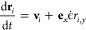
6
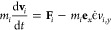
7where ϵ̇ = ∂*v*_*x*_/∂*r*_*y*_ is the shear rate of the external flow and **e**_*x*,*y*_ are the unit vectors along the *x* and *y* directions, respectively. The velocity of the *i*th particle is divided into two parts, that is, the peculiar velocity **v**_*i*_ representing the random thermal motions and the shear flow velocity **e**_*x*_*ϵ̇v*_*i*,*y*_ relating to the external disturbance strength. Specifically, we impose a linear velocity profile in the *x* direction with a constant gradient in the *y* direction, keeping the density of the system constant, by changing the *xy*-tilt factor, *T*_*xy*_, of the triclinic simulation box at a constant shear rate, ϵ̇, as
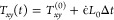
8In eq 8, *T*_*xy*_^(0)^ and *L*_0_ are the initial tilt factor and the original length of the box perpendicular to the shear direction. This can be related to the shear stress of the external shear flow τ_s_ = *μϵ̇*, with μ being the dynamic viscosity of the continuous phase.

### Three-Phase Contact Angle

To estimate the three-phase contact angle, θ_*C*_, for the NPs on the droplets, we calculate the fraction of the spherical NP surface area that is wetted by water,^91^
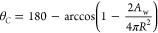
9where *A*_w_ is the area of the NP surface that is wetted by water and *R* is the radius of the NP. The ratio *A*_w_/4*πR*^2^ is obtained by dividing the number of NP surface beads (ap or p) that are wetted by water by the total number of beads on the NP surface. One surface bead is wet by water if a water bead is the solvent bead nearest to it. One standard deviation from the average is used to estimate the statistical uncertainty.

### Interfacial Tension

The interfacial tension γ at the water/oil interface as a function of the NP surface coverage Φ is calculated as^54,56^
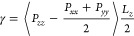
10In eq 10, *P*_*ij*_ is the *ij* element of the pressure tensor, *L*_*z*_ is the simulation box length in the *z* dimension, and the angular brackets denote the ensemble average.

### Self-Diffusion Coefficient

To characterize the self-diffusion coefficient of the NPs at the water/oil interface, we estimate the mean squared displacement of a single NP adsorbed at a planar interface parallel to the *x*–*y* plane. For each particle size, the simulated diffusion coefficient is estimated according to
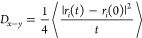
11where *r*_*i*_(*t*) is the position of particle *i* at time *t* on the plane of the interface.

### Gyration Tensor

To measure the evolution of the structural morphology of the emulsion droplet, we estimate the principal components of the gyration tensor,^38,68,92^ which allow the evaluation of the overall shape of the system and reveal its symmetry. Considering the definition for the gyration tensor,
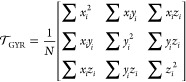
12where the summation is performed over *N* atoms and the coordinates *x*, *y*, and *z* are related to the geometrical center of the atoms, one can define a reference frame where  can be diagonalized:
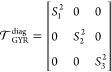
13In eq 13, we follow the convention of indexing the eigenvalues according to their magnitude, *i.e.*, *S*_1_^2^ > *S*_2_^2^ > *S*_3_^2^. We define the radius of gyration *R*_GYR_^2^ ≡ *S*_1_^2^ + *S*_2_^2^ + *S*_3_^2^ and the relative shape anisotropy , and we calculate *R*_GYR_ and κ^2^ using the centers of the water beads.

## Supporting Information Available

Additional information about numerical simulation methods; system characterization (PDF)
