# Supplementary material for: Armored Droplets as Soft Nanocarriers for Encapsulation and Release under Flow Conditions
Source: ACS Nano. 2021 Jul 15;15(7):11406–16. doi: 10.1021/acsnano.1c00955 (PMC8397430; doi:10.1021/acsnano.1c00955)
Supplement: Supplementary file 1 — nn1c00955_si_001.pdf [file nn1c00955_si_001.pdf]

Armored Droplets as Soft Nanocarriers for Encapsulation and Release under Flow Conditions

Supporting Information

François Sicard[*,†,‡] and Jhoan Toro-Mendoza[¶]

†*Department of Physics and Astronomy, University College London, WC1E 6BT London, UK*

‡*Department of Chemical Engineering, University College London, WC1E 7JE London, UK*

¶*Centro de Estudios Interdisciplinarios de la Fisica, Instituto Venezolano de Investigaciones Cientificas, Caracas 1020A, Venezuela*

E-mail: francois.sicard@free.fr

Nanoparticle characteristics

Following previous work,[1–6] the nanoparticles (NPs) are specifically designed to represent Janus silica NPs (particles whose surface shows two distinct wetting properties) at the decane/water interface. The NPs are modelled as hollow rigid spheres with two different diameters, $d_S \sim 3R_c$ and $d_L \sim 6R_c$ for small and large NPs, respectively, with $R_c \sim 0.766$ nm the DPD cutoff distance. Each NP contains polar (p) and nonpolar (ap) DPD beads on its surface and one DPD bead is placed at the NP center for convenience, as shown in Fig. 1a. Hollow models have been used in the literature to simulate NPs, and hollow NPs can also be synthesized experimentally.[7] All types of beads in our simulations have reduced

mass of 1. To cover small and large NPs, 108 and 432 beads are required, respectively, yielding a surface density of ≈ 3.8 beads per R_c^2 on the NP surface.[2] The total number of beads on one NP surface is chosen such that the surface bead density be sufficiently high to prevent other DPD beads (either decane or water) from penetrating the NPs (which would be unphysical), as it has already been explained elsewhere.[8] We use the same surface density for the hydrophobic spherical probe load.

The NP-solvent interaction parameters in the DPD framework, given in the Methods section in the main text, were originally parametrized to reproduce the three-phase contact angle, $\theta_c \sim 85.3^\circ \pm 1.9^\circ$, obtained *via* atomistic molecular dynamics (MD) simulations for one silica Janus NP of diameter $\sim 2R_c$ at the decane/water interface, as explained in previous work.[1–3] In our case, we check that the three-phase contact angles for small and large NPs, $\theta_S \sim 84.1^\circ \pm 2.7^\circ$ and $\theta_L \sim 86.8^\circ \pm 1.1^\circ$, respectively, as shown in Fig. 1b, are in qualitative agreement, within the standard errors, with experimental observations.[9] From the error bars measured, we observe that the small NPs are more sensitive to thermal fluctuations at the interface compared to the large ones, characteristic of the increase of the adsorption energy with the particle radius.[10–12]

To evaluate the diffusion of the small and large NPs at the water/oil interface, we estimate the mean squared displacement (MSD) of a single NP adsorbed at a planar water/oil interface parallell to the $x - y$ plane for increasing simulation lagtime, as shown in Fig. 1c. For each particle size, the MSD is averaged over 5 replicas conducted for 1 μs each, and the simulated diffusion coefficient is estimated accordingly (see Methods section in the main text). We measure $D_S \sim 4.7 \pm 3.1 \times 10^{-7}$ cm^2 s^{-1} and $D_L \sim 1.8 \pm 0.7 \times 10^{-7}$ cm^2 s^{-1}, for small and large NPs, respectively. In particular, large NPs are less diffusive than smaller ones, in qualitative agreement with simulations[12] and experimental observations.[13]

Formation of pocket-like structures

The number of water beads constituting the initial water-in-oil emulsion droplets is fixed to $N_W \approx 3 \times 10^5$. At the beginning of each simulation, the solvent (oil) beads are uniformly distributed within the simulation box. One water droplet of radius $\approx 32\ R_C$ is generated by replacing the oil beads with water beads within the volume of the spherical surface. A number of spherical NPs are placed randomly at the water-decane interface with their polar (nonpolar) part in the water (oil) phase to achieve the desired water-decane interfacial area per NP. The initial configuration obtained is simulated for 10^6 timesteps in order to relax the density of the system and the contact angle of the NPs on the droplet. The system pressure and the three-phase contact angle distributions converged after 5000 simulation steps. Then, we let the system run for an additional 2×10^6 timesteps to generate two additional initial configurations, which allows us to test the reproducibility of the simulations.

To study the surface mechanical instabilities and the collapse mechanisms responsible for the formation of the crater-like depressions at the droplet interface, we follow the numerical protocol discussed by Sicard *et al.* in previous work.[5] The surface area of the droplets is slowly diminished, pumping randomly a constant proportion, *i.e.* 10 percent, of water molecules out of the droplet and letting the system pressure and the three-phase contact angle distribution equilibrate at constant density. To do so, we use the command *fix evaporate*, as implemented in the simulation package LAMMPS,[14] which enables the removal of a given number of atoms from the simulation for a given interval of steps. By slowly, we mean we do not create any hollow volume in the droplet that would strongly drive the system out-of-equilibrium. Doing so, the three-phase contact angle distribution of the NPs evolves sufficiently smoothly when the droplet buckles and becomes nonspherical, thereby preventing particles to be artifactually realeased. This numerical protocol is comparable to a solubilization experiment, where the dispersed phase is slightly soluble in the continuous

phase.[15] By adding a fixed amount of unsatured continuous phase, the volume of the droplets can then be controllably reduced.

To study quantitatively the transition from spherical shrinking to buckling in the uniformly covered droplets, UC_S and UC_L, we follow the evolution of the radial distribution functions, $g_S(r)$ and $g_L(r)$, with r the distance between the center of the NPs, along with the distributions of the three-phase contact angles, θ_S and θ_L, of small and large NPs, respectively. In Fig. 2a, we show the evolution of $g(r)$, as a function of the dimensionless parameter ΔN_W defined in the main text, for UC_S (blue) and UC_L (red). Unlike UC_S where the first peak in $g(r)$ is already present for $\Delta N_W \sim 0.8$ and increases significantly when ΔN_W decreases, we observe the apparition of the first peak in $g(r)$ for UC_L at a later stage ($\Delta N_W \sim 0.72$). This peak increases significantly slower when ΔN_W decreases. This behaviour is representative of the difference in NP interfacial packing as a function of the NP size, with a transition from spherical shrinking to buckling happening when the NP monolayer becomes close to its maximum packing. When the volume of UC_S and UC_L is reduced, $\theta_C^{(S)}$ and $\theta_C^{(L)}$ uniformly evolve from a Gaussian to a skewed unimodal distribution, as shown in Fig. 2b for UC_L, in line with previous work.[5] When the volume of HC_1 or HC_2 is reduced, on the other hand, we observe significant differences in the evolution of the distributions of $\theta_C^{(S)}$ and $\theta_C^{(L)}$, due to heterogeneity in NP size and surface coverage, as shown in Fig. 2c. In particular, the distribution of $\theta_C^{(L)}$ remains similar to the Gaussian distribution observed in the initial configuration (continuous line), while the distributions of $\theta_C^{(S)}$ shows larger variability, characterized with the increase of the asymmetry of the distribution towards lower values of θ_S.

Evolution of the structural morphology of the droplets under flow conditions

As explained in details in the main text and the Methods section, we investigate the dynamical response of the buckled armored nanodroplets HC_1 subjected to shear flow of the surrounding fluid, using the SLLOD algorithm[16,17] coupled with Lee-Edwards periodic boundary conditions.[18] The changes in the structural morphology of the system are characterized with the elongation of the nanodroplet along the deformation axis x, and the squeezing of the crater-like depression along the orthogonal z-direction, as shown in Fig. 3a. In Fig. 3b, we show the probability distribution of the three-phase contact angle, $\theta_C^{(S)}$, for small NPs, at the interface of the structures $HC1_{a,b,c}$ defined in the main text. Within the range of shear rates considered in this work, $\theta_C^{(S)}$ shows a skewed unimodal distribution with a central peak located at the same value as the one measured for both the initial and buckled configurations (shown with continuous lines).

References

1. Fan, H.; Resasco, D.; Striolo, A. Amphiphilic Silica Nanoparticles at the Decane-Water Interface: Insights from Atomistic Simulations. *Langmuir* **2011**, *27*, 5264–5274.

2. Fan, H.; Striolo, A. Nanoparticle Effects on the Water-Oil Interfacial Tension. *Phys. Rev. E* **2012**, *86*, 051610.

3. Luu, X.-C.; Yu, J.; Striolo, A. Nanoparticles Adsorbed at the Water/Oil Interface: Coverage and Composition Effects on Structure and Diffusion. *Langmuir* **2013**, *29*, 7221.

4. Sicard, F.; Striolo, A. Numerical Analysis of Pickering Emulsion Stability: Insights from ABMD Simulations. *Faraday Discuss.* **2016**, *191*, 287–304.

5. Sicard, F.; Striolo, A. Buckling in Armored Droplet. *Nanoscale* **2017**, *9*, 8567–8572.

6. Sicard, F.; Toro-Mendoza, J.; Striolo, A. Nanoparticles Actively Fragment Armored Droplets. *ACS Nano* **2019**, *13*, 9498–9503.

7. Calvaresi, M.; Dallavalle, M.; Zerbetto, F. Wrapping Nanotubes with Micelles, Hemimicelles, and Cylindrical Micelles. *Small* **2009**, *5*, 2191–2198.

8. X-C.Luu,; Yu, J.; Striolo, A. Ellipsoidal Janus Nanoparticles Adsorbed at the Water-Oil Interface: Some Evidence of Emergent Behavior. *J. Phys. Chem. B* **2013**, *117*, 13922–13929.

9. Arnaudov, L.; Cayre, O.; Stuart, M. C.; Stoyanov, S.; Paunov, V. Measuring the Three-Phase Contact Angle of Nanoparticles at Fluid Interfaces. *Phys. Chem. Chem. Phys.* **2010**, *12*, 328–331.

10. Binks, B.; Fletcher, P. Pickering Emulsions Stabilized by Monodisperse Latex Particles: Effects of Particle Size. *Langmuir* **2001**, *16*, 21–41.

11. Jiang, S.; Granick, S. Janus Balance of Amphiphilic Colloidal Particles. *J. Chem. Phys.* **2007**, *127*, 161102.

12. Khedr, A.; Striolo, A. Self-Assembly of Mono- and Poly-Dispersed Nanoparticles on Emulsion Droplets: Antagonistic *vs.* Synergistic Effects as a Function of Particle Size. *Phys. Chem. Chem. Phys.* **2020**, *22*, 22662.

13. Wang, D.; Yordanov, S.; Paroor, H.; Mukhopadhyay, A.; Li, C.; Butt, H.; Koynov, K. Probing Diffusion of Single Nanoparticles at Water–Oil Interfaces. *Small* **2011**, *7*, 3502–3507.

14. Plimpton, S. Fast Parallel Algorithms for Short-Range Molecular Dynamcis. *J. Comput. Phys.* **1995**, *117*, 1–19.

15. Datta, S.; Shum, H.; Weitz, D. Controlled Buckling and Crumpling of Nanoparticle-Coated Droplets. *Langmuir Lett.* **2010**, *26*, 18612–18616.

16. Evans, D. J.; Morriss, G. P. Non-Newtonian Molecular Dynamics. *Comput. Phys. Rep.* **1984**, *1*, 297.

17. Evans, D.; Morriss, G. Nonlinear-Response Theory for Steady Planar Couette Flow. *Phys. Rev. A* **1984**, *30*, 1528.

18. Lees, A.; Edwards, S. F. The Computer Study of Transport Processes under Extreme Conditions. *J. Phys. C* **1972**, *5*, 1921.

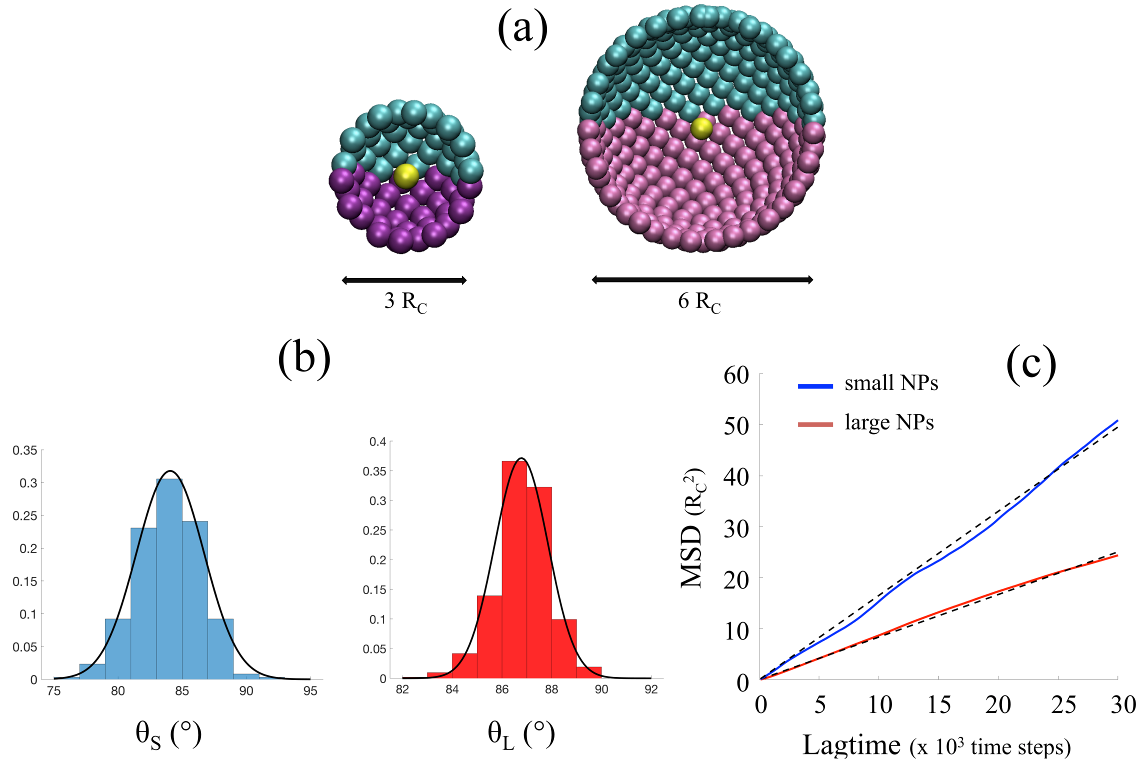

Figure 1: (a) Cross sectional view of the small (left panel) and large (right panel) spherical NPs simulated in this work. Cyan, purple and gold spheres represent the nonpolar (ap), polar (p), and NP center beads, respectively. Small and large NPs are covered with 108 and 432 beads,respectively, corresponding to a surface density of ~ 3.8 beads per R_c^2 on the NP surface. The fractions of nonpolar and polar beads on the NP surface are identical. (b) Probability distributions of the three-phase contact angles θ_S and θ_L for small (S) and large (L) NPs, respectively. The probability distributions is fitted with Gaussian distributions of means $\mu_S \sim 84.1°$ and $\mu_L \sim 86.8°$, and standard deviations $\sigma_S \sim 2.7°$ and $\sigma_L \sim 1.1°$, as shown with continuous lines. (c) MSD as a function of simulation lagtime for small and large NPs measured at the water/oil planar interface.

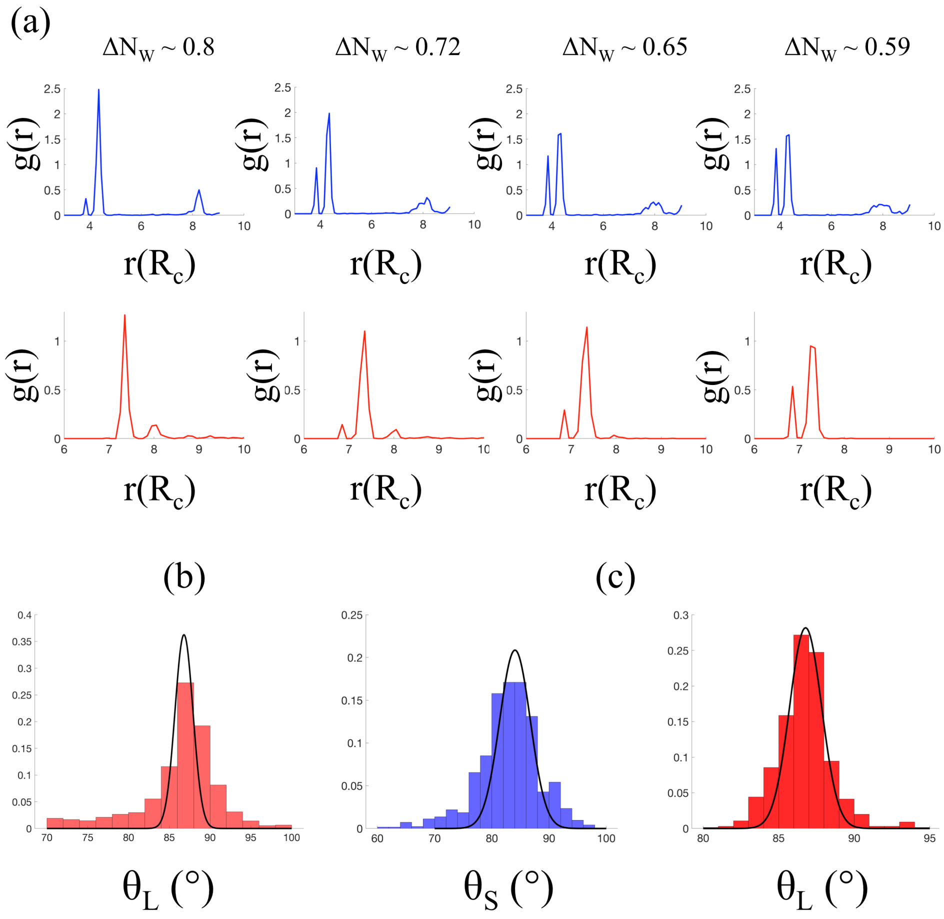

Figure 2: (a) Evolution of the NP radial distribution function, $g(r)$, as a function of the dimensionless parameter ΔN_W, defined in the main text, when the droplet is uniformly covered with small (top panel) and large (bottom panel) NPs. (b) Probability distribution of the three-phase contact angle of large NPs, θ_L, at the interface of UC_L, when $\Delta N_W \sim 0.35$. The initial Gaussian distribution, fitted with continuous line, is shown for comparison. (c) Probability distributions of the three-phase contact angle of θ_S and θ_L, for small and large NPs, respectively, at the interface of HC_1, when $\Delta N_W \sim 0.35$. The initial Gaussian distributions, fitted with continuous lines, are shown for comparison.

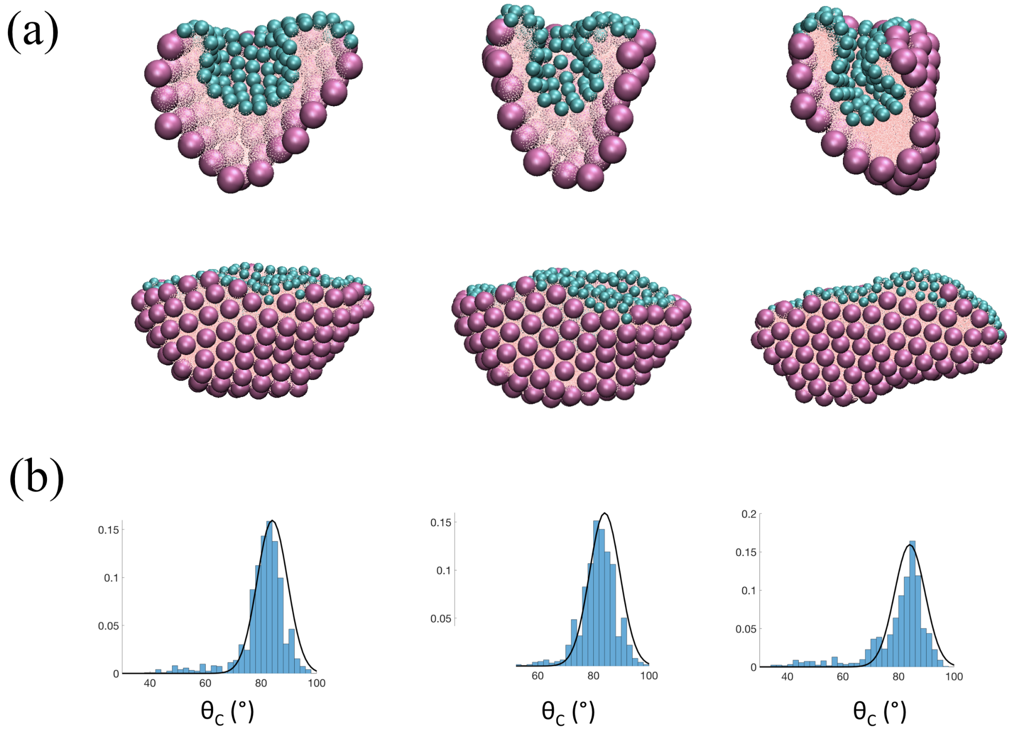

Figure 3: (a) Representative cross-view (top) and side-view (bottom) of HC1$_{a,b,c}$ (from left to right) obtained after the relaxation of the system ($t \sim 1.2\ \mu s$). Cyan and purple spheres represent the small and large Janus NPs, respectively. Pink spheres represent water beads. The oil molecules surrounding the system are not shown for clarity. (b) Corresponding distributions of the three-phase contact angle, $\theta_C^{(S)}$, for small NPs, at the interface of the structures HC1$_{a,b,c}$.
